# Superior Anti-Tumor Response After Microbeam and Minibeam Radiation Therapy in a Lung Cancer Mouse Model

**DOI:** 10.3390/cancers17010114

**Published:** 2025-01-01

**Authors:** Narayani Subramanian, Aleksandra Čolić, Marina Santiago Franco, Jessica Stolz, Mabroor Ahmed, Sandra Bicher, Johanna Winter, Rainer Lindner, Susanne Raulefs, Stephanie E. Combs, Stefan Bartzsch, Thomas E. Schmid

**Affiliations:** 1 Department of Radiation Oncology, TUM School of Medicine and Health and Klinikum rechts der Isar, University Hospital of the Technical University of Munich, Ismaninger Straße 22, 81675 Munich, Germany; narayani.subramanian@tum.de (N.S.); aleksandra.colic@helmholtz-munich.de (A.Č.); marina.franco@tum.de (M.S.F.); jessica.stolz@tum.de (J.S.); mabroor.ahmed@helmholtz-munich.de (M.A.); sandra.bicher@tum.de (S.B.); johanna.winter@tum.de (J.W.); susanne.raulefs@tum.de (S.R.); stephanie.combs@tum.de (S.E.C.); 2 Institute of Radiation Medicine (IRM), Helmholtz Zentrum, 85764 München, Germany; rainer.lindner@helmholtz-munich.de

**Keywords:** microbeam radiation therapy, minibeam radiation therapy, progression-free survival, tumor growth delay, lung cancer, spatially fractionated radiation therapy

## Abstract

Objectives: The present study aimed to compare the tumor growth delay between conventional radiotherapy (CRT) and the spatially fractionated modalities of microbeam radiation therapy (MRT) and minibeam radiation therapy (MBRT). In addition, we also determined the influence of beam width and the peak-to-valley dose ratio (PVDR) on tumor regrowth. Methods: A549, a human non-small-cell lung cancer cell line, was implanted subcutaneously into the hind leg of female CD1*-Foxn1^nu^* mice. The animals were irradiated with sham, CRT, MRT, or MBRT. The spatially fractionated fields were created using two specially designed multislit collimators with a beam width of 50 μm and a center-to-center distance (CTC) of 400 μm for MRT and a beam width of 500 μm and 2000 μm CTC for MBRT. Additionally, the concept of the equivalent uniform dose (EUD) was chosen in our study. A dose of 20 Gy was applied to all groups with a PVDR of 20 for MBRT and MRT. Tumor growth was recorded until the tumors reached at least a volume that was at least three-fold of their initial value, and the growth delay was calculated. Results: We saw a significant reduction in tumor regrowth following all radiation modalities. A growth delay of 11.1 ± 8 days was observed for CRT compared to the sham, whereas MBRT showed a delay of 20.2 ± 7.3 days. The most pronounced delay was observed in mice irradiated with MRT PVDR 20, with 34.9 ± 26.3 days of delay. Conclusions: The current study highlights the fact that MRT and MBRT modalities show a significant tumor growth delay in comparison to CRT at equivalent uniform doses.

## Simple Summary

Radiation therapy is applied to approximately half of all cancer patients worldwide. For some tumors, its success is still limited due to radiation-sensitive organs at risk in the tumor’s proximity and the occurrence of severe side effects in the surrounding normal tissue. Spatial fractionation is an innovative strategy that substantially reduces radiation-induced damage to normal tissues while effectively maintaining tumor control rates, leading to an increased therapeutic window. The aim of the present study was to determine the tumor growth delay in an in vivo xenograft mouse model, comparing novel spatial fractionated treatment modalities with conventional radiotherapy. We confirmed a significant reduction in tumor regrowth following all radiation modalities, with microbeam radiation therapy showing the most pronounced growth delay, followed by minibeam radiation therapy.

## 1. Introduction

Lung tumors are a driving cause of cancer incidence and mortality worldwide. The most recent Global Cancer Observatory (GLOBOCAN) figures indicate that 2,480,675 new instances of lung cancer were diagnosed worldwide in 2022, making it the cancer with the highest incidence. Besides its high incidence, lung cancer still has high mortality rates, with an estimated 1,817,469 deaths in 2022 alone, which constituted approximately 16.8% of all cancer-related deaths worldwide [1]. The high mortality is attributed to a poor prognosis [2], with the 5-year survival for advanced-stage lung cancer ranging from 0% to 10%, whereas for early stages, it is considerably higher, at 68% [3].

Radiation therapy (RT), used with both curative and palliative intent, has shown to improve the overall median survival in clinical studies, with promising results seen in patients with stage II/III non-small-cell lung cancer (NSCLC) [4]. However, acute side effects such as pneumonitis and late side effects such as lung fibrosis are very common, especially with central lesions [5]. The implementation of innovative radiation procedures that reduce normal tissue toxicity is important to improve therapeutic outcomes for patients.

One novel pre-clinical method of irradiation called microbeam radiation therapy (MRT), developed by Daniel Slatkin and colleagues in 1992 [6], has been investigated over the last few decades. MRT is a form of spatially fractionated radiation therapy (SFRT), which consists of highly collimated, quasi-parallel 25–100 μm wide arrays of X-ray beams. This leads to high-dose “peak” regions and low-dose “valley” regions in the target. The low valley dose and high peak-to-valley dose ratio (PVDR) of MRT can substantially lower normal tissue damages while simultaneously maintaining or even increasing tumor control [7,8,9]. For lung tumors, MRT with peak doses of up to 400 Gy has been shown to effectively decrease tumor size in a mouse model when compared to conventional broadbeam radiation [9]. The remarkable results from this study conducted by Schültke et al. in 2021 also revealed the absence of fibrosis formation following MRT, even after the administration of very high peak doses. This is a promising finding, as MRT can help preserve normal tissue in patients who require intense, high doses of radiation.

Minibeam radiation therapy (MBRT) is another innovative treatment method and utilizes arrays of sub-millimeter (400–700 µm) planar X-ray beams or proton beams [10]. Developed in 2006, MBRT was designed to overcome some of the shortcomings of MRT while still maintaining a high therapeutic index. Also, MBRT has been positively correlated with improved tumor control while reducing normal tissue tolerance. Deman et al. (2012) and Prezado et al. (2012) [11,12] have shown excellent tumor control in glioma-bearing rats after exposure to 123 Gy MBRT (at 1 cm depth in the brain). Due to its larger beam and dose profiles, MBRT has an advantage over MRT in that its dose distribution pattern is not affected by heart pulsations [13]. This is especially important in irradiations concerning the lungs.

Despite advances in the field of SFRT, clinical translation is still an ongoing challenge due to two main factors.

1. The generation of X-ray microbeams and minibeams for pre-clinical research requires third-generation synchrotron sources. In order to make the technology more accessible for research, several attempts have been made to build compact X-ray sources enabling MRT and MBRT. In this study, we used a XenX (X-Strahl, Camberley, UK) equipped with two self-designed tungsten collimators [14].
2. The radiobiological mechanisms of MRT and MBRT are not understood. Existing hypotheses include non-targeted effects, which include bystander effects, oxidative damage by reactive oxygen species (ROS), vascular effects, and immune effects [10].

Here, we present an *in vivo* investigation of the influence of both beam width and PVDR on tumor growth delay (A549, human NSCLC) at the same irradiation cabinet. In order to narrow down the underlying mechanisms of MRT and MBRT, we chose an immunocompromised mouse model to exclude the effects of the immune system. We established the xenograft tumor model for our study, irradiated the mice with sham, conventional broadbeam (CRT), MRT, or MBRT (both with a PVDR of 20), and compared the tumor growth delay between the various modalities. Subsequently, we collected blood from the tail veins for the quantitative polymerase chain reaction (qPCR) analysis of genes involved in free radical scavenging.

## 2. Materials and Methods

### 2.1. Animal Studies

All animal experiments were approved by the local government authorities (Regierung von Oberbayern, Munich, Germany) with the project license ROB-55.2-2532.Vet_02-18-170 and performed according to project guidelines. Eight- to ten-week-old female CD1-*Foxn1^nu^* mice (Charles River Laboratories, Research Models and Services GmbH, Sulzfeld, Germany) were allowed to acclimate for one week before the start of the experiments. The mice were housed in individually ventilated cages at a 12 h light/dark cycle and had access to food and water *ad libitum*.

### 2.2. Tumor Cell Injection

In order to avoid tumor cell rejection, all mice received a total body irradiation two to three days prior to tumor cell injection using the X-Strahl CIX3 cabinet X-ray irradiator (X-Strahl Limited, Camberley, UK) with a dose of 4 Gy [15]. Following total body irradiation, the mice were subcutaneously administered a prophylactic antibiotic Cefovecin (Convenia, Zoetis Belgium SA, Parsippany, NJ, USA) with a dose of 1 µL/g body weight.

For the tumor cell injection, the mice were anesthetized with isoflurane. A549 (human NSCLC) cells suspended in Matrigel^®^ (Corning Inc., Corning, NY, USA) were injected subcutaneously into the right hind leg of the mice, between the ankle and the knee fold. Three million cells in 50 µL of Matrigel^®^ were injected per mouse. The tumor volumes were measured using a caliper, calculated with the ellipsoid equation (π/6 × length × breadth^2^), and irradiated when the volumes reached 60–100 mm^3^.

### 2.3. Setup and Radiation Therapy

**Figure 1:**
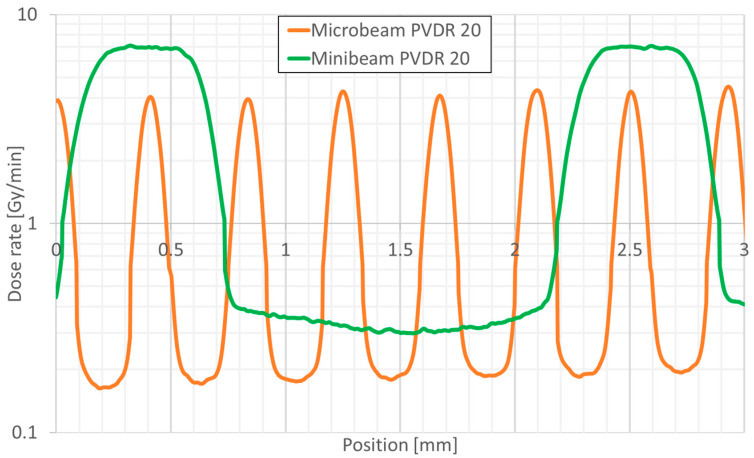
Dose profiles of MRT and MBRT with a peak-to-valley dose ratio 20 generated with film dosimetry.

All irradiations were performed using the XenX small-animal irradiation system (X-Strahl Limited, Camberley, UK) equipped with two custom-made tungsten microbeam and minibeam multislit collimators. The device was equipped with a motorized sample stage and a mouse holder. For MRT, the collimator beam width was 50 µm, and the center-to-center (CTC) distance between two consecutive peaks was 400 µm. For MBRT, the collimator beam width was 500 µm and the CTC was 2000 µm. The treatment field always had a size of 10 × 10 mm^2^, covering 5 minibeams and 25 microbeams. A total of 36 mice were randomly assigned into the following four groups with 9 mice per group: sham controls, conventional broadbeam, microbeam PVDR 20, and minibeam PVDR 20. The concept of an equivalent uniform dose (EUD) [16] was applied for dose calculation (alpha = 0.4460 and beta = 0.0115 for A549) and an equivalent dose of 20 Gy was administered to all irradiation groups. All irradiations were carried out with a 225 kVp X-ray spectrum filtered by 1 mm aluminum. The peak doses for the MRT and MBRT groups were 470 ± 2 Gy and 418 ± 2 Gy, respectively. The valley doses for the MRT and MBRT groups were 23.5 ± 2 Gy and 20.9 ± 2 Gy, respectively. Different PVDRs were achieved by varying the distance between the collimator and the mouse holder with polymethyl methacrylate (PMMA) scatter material (15 mm PMMA for MBRT and 8 mm PMMA for MRT). The dose profiles for MRT and MBRT are shown in Figure 1.

The mice were anesthetized with isoflurane, and Bepanthane^®^ eye cream (Bayer, Leverkusen, Germany) was applied to prevent drying of the eyes during treatment. The mice were immobilized in the mouse holder inside the XenX, and isoflurane was maintained at volume concentrations between 1.3 and 2% during treatment, to keep a breathing frequency of approximately 1 Hz. Dose delivery was verified using film dosimetry with GAFCHROMIC™ EBT-3 films (Ashland Global Holding Inc., Kentucky, DE, USA). Furthermore, the temperature inside the Xen-X was maintained at 28 °C, and the mice were monitored externally during irradiation using a camera placed inside the cabinet. Following radiation therapy, the mice were removed from the mouse stage, observed for an additional hour, and returned to their cages.

### 2.4. Tumor Growth Measurements

The tumors were measured three times a week and weighed at least once a week following irradiation. The tumors were always measured by a single operator throughout the course of this study. The tumor growth time was defined as the time a tumor needed, after treatment, to reach three times the initial volume V_0_ (volume at the time of irradiation). The tumor growth delay was calculated as the difference in growth time between treated tumors and untreated controls [17]. Animals were euthanized when the tumor exceeded three times its initial volume. The regrowth process of the tumors after treatment was modeled as the sum of two exponential functions:

(1) $$f\left(t\right)=\frac{V\left(t\right)}{V_{0}}=A_{1}e^{-\alpha_{1}t}+A_{2}e^{\alpha_{2}t}$$

where *A*_1_ + *A*_2_ = 1 and *A*_1_, *A*_2_, *α*_1_, and *α*_2_ > 0. The component $A_{1}e^{-\alpha_{1}t}$ describes the fraction of cells in a tumor that undergo exponential shrinkage due to cell killing by irradiation, and $A_{2}e^{\alpha_{2}t}$ accounts for the fraction of surviving neoplastic cells that undergo exponential proliferation. The function was used to estimate the start of the regrowth process after irradiation by fitting the recorded tumor volumes and calculating the global minimum of the curve obtained for each treatment group. The regrowth process was said to start the day after the smallest tumor volume was highlighted as the global minimum. The progression-free survival (PFS) probability was defined as the time between irradiation and the start of the regrowth process. The start of the regrowth process was considered a termination endpoint for PFS probability. The PFS was plotted using a Kaplan–Meier curve, which is commonly used to analyze time–event relationships [18].

### 2.5. Blood Collection, RNA Isolation, and Quantitative Real-Time Polymerase Chain Reaction (qPCR)

Blood was collected by cannulation from the lateral caudal veins 24 h post irradiation. The blood collection did not exceed 0.6 mL/kg body weight. The blood was collected in RNAprotect animal blood tubes (Qiagen, Hilden, Germany). Total RNA isolation and cDNA synthesis was carried out using the RNeasy protect animal blood kit (Qiagen, Hilden, Germany) and the RT^2^ first strand kit (Qiagen, Hilden, Germany) as per the manufacturers’ guidelines. qPCR was performed for the two genes, superoxide dismutase 1 (SOD1) and glutathione (GSH) peroxidase 1 (GPx1), whose primers were purchased from the Quantitect^®^primer assay (Qiagen, Hilden, Germany) and run according to the manufacturer’s guidelines. Melting curves were used to monitor and eliminate nonspecific amplifications, and the relative quantification of the genes of interest was computed using the 2^−ΔΔCT^ method.

### 2.6. Statistical Analysis

The Kaplan–Meier survival analysis was used to estimate the progression-free survival (PFS) for each treatment arm. Comparison between the treatment groups and untreated controls, as well as between treatment groups with respect to the PFS, was carried out using a stratified log-rank (Mantel–Cox) test at a 5% level of significance (two-sided). For the tumor growth delay, the mean values for each treatment group with their corresponding standard deviations were calculated, along with a 95% confidence interval. A two-tailed unpaired *t*-test was performed to compare different treatment groups, and *p*-values of <0.05 were considered statistically significant. All analyses were performed using Prism version 9.5.0 (GraphPad Software, Boston, MA, USA, www.graphpad.com, accessed on 7 July 2024). The tumor growth delay graph was generated using Python version 3.13.

## 3. Results

### 3.1. Tumor Growth Delay

The parameters in Equation (1) were determined by minimizing the square distances between f(t) and measurements of V and V_0_ using the generalized reduced gradient non-linear solver in Microsoft Excel for each mouse. Following this, the standard deviation, 95% confidence interval, and Student’s *t*-test were performed to determine significant differences between the different groups compared to the controls (Table 1).

**Figure 2:**
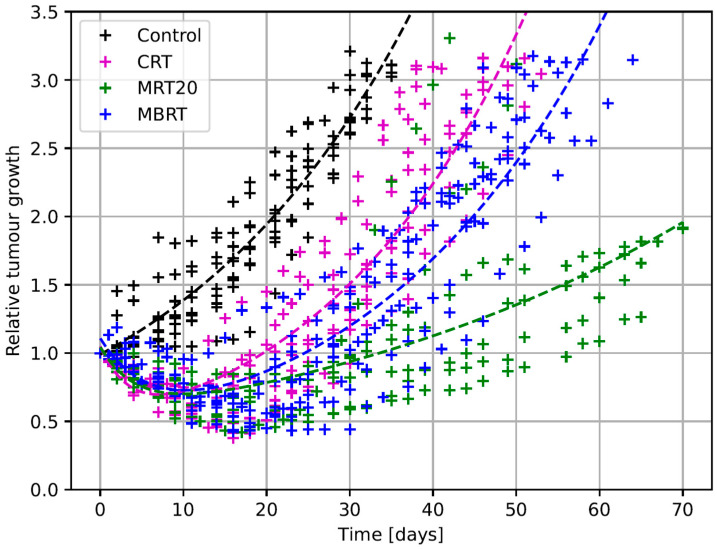
Tumor growth of all experimental groups normalized to the controls. CRT: Broadbeam; MBRT: minibeam radiation therapy; and MRT20: microbeam radiation therapy with peak-to-valley dose ratio 20. The “+” represents single data points, while the dashed line represents the fit of the average tumor volume of survivors at a given time point.

A comprehensive comparison of the growth delay is shown in Figure 2. Control tumors reached the endpoint of three times the initial tumor volume in 33 ± 5 days, while BB CRT showed a significant (*p* = 0.0004) growth delay of 11.1 ± 8.0 days. Significant growth delays were also observed for the spatially fractionated groups. The MRT PVDR 20 group showed the strongest tumor growth delay of 34.9 ± 26.3 days (*p* = 0.0002), followed by the MBRT, with a delay of 20.2 ± 7.3 days (*p* = 1.6× 10^−6^).

### 3.2. Progression-Free Survival

**Figure 3:**
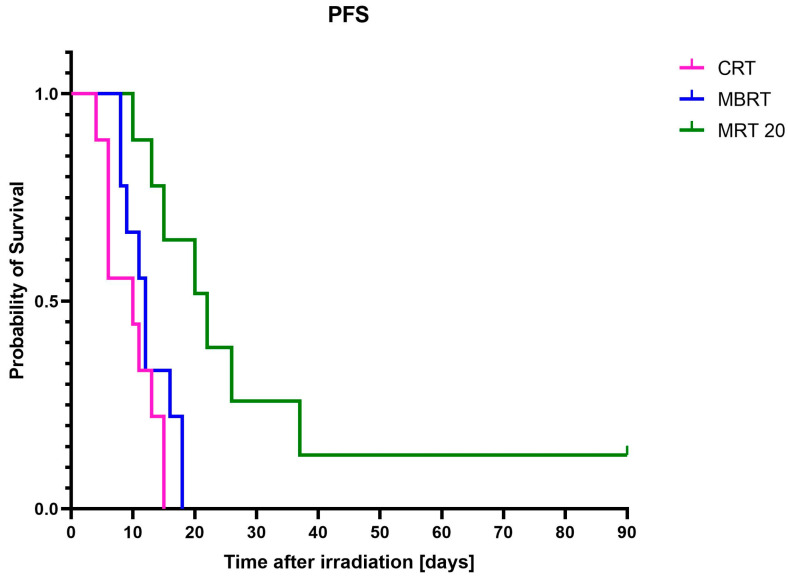
Progression-free survival (PFS) analysis of all experimental groups. CRT: conventional; MBRT: minibeam radiation therapy; and MRT 20: microbeam radiation therapy with a peak-to-valley dose ratio 20. The graph was generated using Prism v9.5.0.

The follow-up time since randomization for all mice was 90 days after irradiation or until the endpoint (three times the initial tumor volume) was reached. The MRT 20 group showed the highest PFS (Figure 3) with two mice out of nine showing signs of controlled tumors with no regrowth for about 90 days, after which they were euthanized according to the project guidelines.

A log-rank (Mantel–Cox) test was performed to compare the survival distribution between the different experimental groups (other than the controls). The survival distributions (Table 2) were significant between MRT and all other irradiation groups with *p* = 0.0018 and 0.0065 for CRT and MBRT, respectively. The differences were not statistically significant between MBRT and CRT.

### 3.3. Expression of GPx1 and SOD1 in Different Subgroups

**Figure 4:**
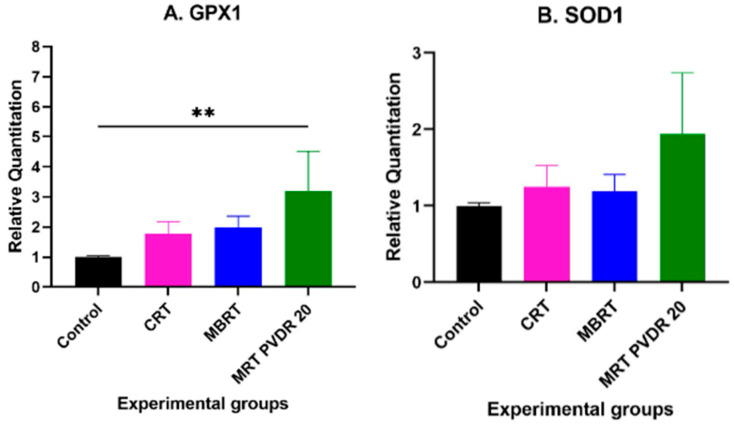
GPx1 levels (**A**) and SOD1 levels (**B**) measured in whole blood among all experimental groups 24 h post irradiation. Results are expressed as mean ± SD (*n* = 4) (** *p* < 0.05). The graph was generated using Prism v9.5.0.

The expression levels of GPx1 and SOD1 were normalized to three endogenous control genes—GAPDH, B2M, and β-actin. The average of all biological replicates of each treatment group’s expression was normalized to that of the control group. The GPx1 levels were found to be elevated in all experimental groups, with significantly higher expression in the MRT 20 group (*p* < 0.05) (Figure 4A). The SOD1 levels were about two-fold elevated in the MRT PVDR 20 group. Broadbeam-irradiated groups and minibeam-irradiated groups showed similar levels to the control group (Figure 4B).

## 4. Discussion

RT is the sole treatment option for lung cancer patients with inoperable tumors and for those with tumor recurrence following surgery [19]. The enhanced normal tissue-sparing effects of MBRT and MRT [20,21] compared to CRT were a key motivation behind our present study investigating these modalities. To the best of our knowledge, this is the first study that directly compares the effectiveness of both microbeams and minibeams with CRT using the same irradiation device.

In the current study, tumors developed in all 36 mice (100% tumor take rate) and showed exponential tumor growth. There was a statistically significant delay in tumor regrowth in all experimental groups compared to sham-irradiated mice. One approach explaining the effectiveness of SFRT includes damage to the blood vessels, which is known to have a dose threshold. It has been shown that peak doses of 400 Gy and above are required to cause irreversible vascular blockage [22]. Therefore, PVDR is an important dosimetric parameter that can influence the biological effectiveness of SFRT response.

Compared to unirradiated controls, CRT showed a significant tumor growth delay, which was even more pronounced in the SFRT groups. The tumor growth delay was much more prominent in microbeams compared to minibeams, with a difference of over 15 days. This suggests a significant influence of beam width on the tumoricidal effect of SFRT. Our results are in line with previously reported studies [23] in glioma-bearing rats where the median survival time (MST) was significantly higher in the 50 μm MRT group (53 days vs. 18 days for the sham), which fits our study (median PFS of 22 days for MRT animals vs. 10 days for CRTBB). It should be noted that we do not control the number of beams hitting the tumor. Especially for the minibeams, the volume fraction covered by the peak dose will vary statistically within the group. However, a systematic bias is not to be expected.

Tumor growth delay was also investigated in other entities with MRT. In the study by Dilmanian et al. [7], EMT-6, a murine mammary carcinoma, was irradiated with 90 μm wide microbeams (CTC 300 μm, peak entrance doses ranging from 800 Gy to 1900 Gy). Higher peak doses showed a 100% tumor control rate compared to lower peak doses, which showed rates of up to 25%, while CRT showed up to 75% tumor control rate, albeit with a high grade of toxicity, which manifested as tissue necrosis, epilation, and swelling. This is in line with the present study, where few CRT- and MRT-irradiated mice (*n* = 7) displayed redness and inflammation in the irradiated area, which subsequently regressed in 3 to 5 days, following the application of Bepanthane ointment. Minibeam-irradiated mice did not show any normal tissue side effects. It has been shown that very high peak doses (>1000 Gy) in SFRT can cause severe normal tissue toxicity [7,23,24]. However, recent studies [25] have demonstrated that the valley dose is the most relevant parameter with respect to acute toxicity. Optimal tumor control requires the use of higher peak doses with the valley dose staying below normal tissue tolerance. In a study by Griffin et al. [26], 50 μm wide microbeams with a CTC of 200 μm with peak entrance doses of 150 Gy were found to be the most effective in delaying tumor growth in a radioresistant murine mammary carcinoma model in comparison to 500 μm wide minibeams with a CTC of 2000 μm. This finding is supportive of the results in our study, given that the irradiation conditions for MRT and MBRT for both experiments are very similar.

We found a three-fold increase in GPx1 levels 24 h post MRT irradiation compared to the sham controls (Figure 4A). GPx1 increase following irradiation is not well characterized. Some studies have shown an inactivation of GPx1 following irradiation [27,28], while another study has shown an upregulation in murine lung tissues [29]. However, previous research has shown that lung cancer cells frequently overexpress endogenous antioxidants, which is linked to a poorer prognosis [30]. GPx1 can also act as an oncogene by regulating proliferation, apoptosis, and migration, among other tumor-promoting effects. The overexpression of GPx1 has also been negatively correlated with overall survival in several types of cancers such as breast, gastric, glioma, and leukemia [31]. SOD1 levels following irradiation were not statistically significant in any of the groups (Figure 4B). However, the MRT group showed a marginal increase 24 h post irradiation. GPx and SOD are enzymes involved primarily in the detoxification of reactive oxygen species (ROS). Detrimental levels of ROS can directly inflict oxidative stress in irradiated cells, which can activate oxidative phosphorylation (OXPHOS) pathways and subsequently activate cellular antioxidant enzymes. Elevated ROS levels and the subsequent activation of antioxidant enzymes are a key part of radiation-induced adaptive response [32]. Further studies are needed to characterize the kinetics of ROS scavengers following SFRT.

According to the mentioned studies, MRT and MBRT have been positively correlated to a greater therapeutic index in comparison to conventional broadbeam irradiation. This could be attributed to several reasons. Microbeams show a 90–10% dose fall-off gradient between tissue slices receiving the peak and valley doses. As a result, the radiotoxic dose is contained inside a very small area. Furthermore, compared to what is provided by a single broadbeam, spatial fractionation produces a substantially larger specific contact area between peak and valley zones [33,34]. Tissues with an extremely high dose in peak regions can be replaced by significantly less-harmed tissues in the valleys due to this wider contact surface [33].

The superior effect of MRT and MBRT over CRT also holds true for several other studies like Trapetti et al. [35] and their study on the growth delay of melanoma, in which MRT showed effective tumor growth delay and tumor shrinkage of up to 85.7% compared to conventional broadbeam irradiation. Miura et al. [36] used a mouse model of aggressive murine SCCVII squamous cell carcinomas transplanted subcutaneously into the left thighs of female C3H mice. They demonstrated that MRT irradiations (peak width 35 μm; CTC 200 µm; 625 Gy or 884 Gy peak dose and peak width 70 μm; CTC 200 µm; 442 Gy peak dose) produced a superior palliative effect than BB irradiations (25 or 35 Gy).

In our study, the SFRT groups showed a superior anti-tumor growth delay response compared to conventional RT. This study suggests the importance of both peak doses and PVDR when it comes to the clinical translation of SFRT.

## 5. Conclusions

This study shows that MBRT and MRT at a PVDR of 20 show an increased tumor growth delay compared to conventional treatment at an equal equivalent uniform dose in the virtual absence of an intact immune system. This study was the first of its kind to irradiate both microbeams and minibeams in the same irradiation cabinet, independent of synchrotrons. Tumor growth delay studies correspond to palliative radiation therapy and are extremely important for improving the quality of life in patients with advanced-stage cancer. The tumor growth delay effects exhibited by MBRT and MRT in this study show a promising transition from pre-clinical studies to clinical studies.

## Figures and Tables

**Table 1:** The *t*-test values between different experimental groups normalized to the control group. A *p*-value of <0.05 was considered statistically significant.

Groups	Tumor Growth Delay (Days) ± SD	95% CI	*p*-Value
Control	-	-	-
CRT	11.1 ± 8.0	3.6	0.0004
Minibeam PVDR 20	20.2 ± 7.3	3.2	0.000001
Microbeam PVDR 20	34.9 ± 26.3	18.8	0.0002

**Table 2:** Log-rank (Mantel–Cox) test between the experimental groups. A *p*-value of <0.05 was considered statistically significant.

Experimental Groups	*p*-Value	Significance
MRT vs. CRT	0.0018	yes
MRT vs. MBRT	0.0065	yes
MBRT vs. CRT	0.1416	no

## Data Availability

The original contributions presented in this study are included in the article. Further inquiries can be directed to the corresponding authors.
